# The Biological Role and Clinical Significance of BECLIN-1 in Cancer

**DOI:** 10.3390/ijms26199380

**Published:** 2025-09-25

**Authors:** Chinmay Maheshwari, Andrea Castiglioni, Uthman Walusimbi, Chiara Vidoni, Alessandra Ferraresi, Danny N. Dhanasekaran, Ciro Isidoro

**Affiliations:** 1 Laboratory of Molecular Pathology, Department of Health Sciences, Università del Piemonte Orientale, Via Solaroli 17, 28100 Novara, Italy; chinmay.maheshwari@uniupo.it (C.M.); a.castiglioni91@gmail.com (A.C.); uthman.walusimbi@uniupo.it (U.W.); chiara.vidoni@med.uniupo.it (C.V.); alessandra.ferraresi@med.uniupo.it (A.F.); 2 Stephenson Cancer Center, The University of Oklahoma Health Sciences Center, Oklahoma City, OK 73104, USA

**Keywords:** autophagy, BECLIN-1, tumor suppressor gene, apoptosis, epigenetics, alternative splicing, prognosis

## Abstract

BECLIN-1 is a multidomain protein that, through dynamic interaction with a variety of partners, controls autophagy and apoptosis, two processes dysregulated in cancer cells, thus playing a crucial role in cell fate. Although mutations in the *BECN1* gene are rare in cancer, its frequent monoallelic deletion contributes to spontaneous cancer initiation by impairing autophagy, establishing it as a haploinsufficient tumor suppressor gene. The expression and activity of BECLIN-1 are further modulated by epigenetic mechanisms, alternative splicing, post-translational modifications, and alternative partner interactions. These layers of regulation critically affect the autophagy response, with an impact on cell proliferation, motility, and resistance to multiple stress stimuli. In this review article we outline the structural and functional properties of BECLIN-1 and discuss how its altered expression and protein–protein interactions can be harnessed for diagnostic and therapeutic purposes in cancer.

## 1. Introduction

Autophagy is a lysosome-dependent degradation mechanism that eliminates damaged or superfluous cellular structures with recovery of material for biosynthesis. This catabolic process is essential for maintaining homeostasis and cellular survival. The term “autophagy” was introduced in 1963 by Christian De Duve, the scholar who won the Nobel Prize in 1974 for the discovery of lysosomes, since it implies the “eating of self” cellular components [1,2]. The physiological importance of this catabolic process was neglected for decades until the early 1990s, when its underlying molecular mechanisms were unveiled in the budding yeast *Saccharomyces cerevisiae*. Yoshimori Oshumi, who was awarded the Nobel Prize in 2016 for having cloned 15 autophagy (ATG) genes, and other researchers, including Daniel Klionsky and Michael Thumm, laid the foundations for modern autophagy research [2]. Soon after, the mammalian orthologs of the yeast ATG genes had been identified, making it possible to study and better appreciate the pathophysiological relevance of this process also in mammalian organs. One of the earliest identified yeast-like mammalian autophagy master regulators was BECLIN-1, a coiled-coil protein discovered by Beth Levine’s lab as an interactor of the anti-apoptotic BCL-2 [3]. Dr. Beth Levine (1960–2020) was a highly influential scientist whose groundbreaking research in autophagy, with the discovery of BECLIN-1 (and BECLIN-2, see Appendix A), profoundly advanced our understanding of cell biology and disease pathogenesis, with major implications for cancer and neurodegenerative disorders.

**Figure 1:**
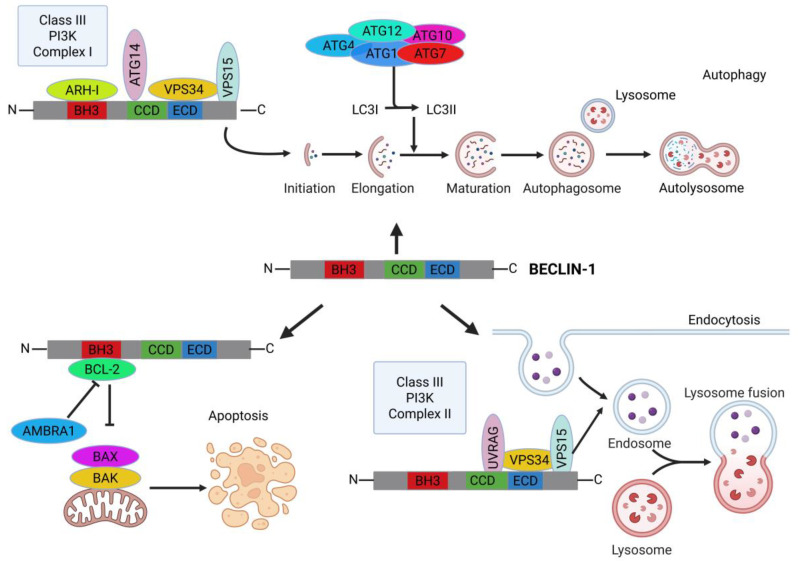
BECLIN-1 as a central regulator of cell survival/cell death balance and endocytic trafficking. At the start of autophagy, BECLIN-1 forms a class III PI3K complex 1, which is involved in autophagosome formation and the recruitment of other autophagy proteins, allowing the elongation, maturation, and cargo incorporation into the autophagosome. Additionally, BECLIN-1, as part of the class III PI3K complex 2, plays a role in endocytic trafficking by regulating the fusion and maturation of early into late endosomes. BECLIN-1 also plays a key role in apoptosis, where it sequesters the anti-apoptotic protein BCL-2 through its BH3 domain, thereby releasing its inhibitory effect on apoptosis. Abbreviations: Activating molecule in BECLIN-1-regulated autophagy protein 1, AMBRA1; Aplasia Ras homologue member I, ARH-I; Autophagy-related gene, ATG; B-cell lymphoma 2, BCL-2; BCL-2 homologous antagonist/killer; BAK; BCL-2 homology 3, BH3; BCL-2-associated X protein, BAX; Coiled-coil domain, CCD; Evolutionary conserved domain, ECD; Light chain 3, LC3; Phosphatidylinositol 3-kinase, PI3K; UV radiation resistance-associated gene, UVRAG; Vacuolar protein sorting, VPS.

Autophagy is primarily a pro-survival adaptive mechanism that aims to restore cell homeostasis in response to endogenous and environmental stress stimuli, and as such, its dysregulation is causally linked to a wide range of diseases [4]. Basal autophagy safeguards genomic stability and cell homeostasis by removing damaged organelles and protein aggregates and ensuring macromolecular and organelle turnover [5]. Defective autophagy can result in the accumulation of cellular damage, potentially leading to malignant transformation as an adaptation to chronic insults [5,6]. Conversely, hyperactivation of autophagy leads to excessive degradation of vital organelles (e.g., mitochondria, endoplasmic reticulum, and ribosomes), causing autophagic cell death [7]. In cancer, autophagy exerts a dual role: it can act as a tumor suppressor, preventing cell transformation and tumor growth, or as a tumor promoter by enabling resistance to stress conditions [8]. For example, in the hypoxic niche, dysregulated autophagy may turn to help tumor cells to survive in harsh metabolic conditions, leading to the persistence of dormant cells resistant to immune response and chemotherapy [9]. The mechanistic pivot underlying these context-dependent outcomes lies in BECLIN-1 as the core component of two alternative complexes, the class III PI3K complex and the BCL-2-BECLIN-1 complex [10,11], that determine whether cells undergo survival or cell death in response to stress (Figure 1).

Despite three decades of research since the BECLIN-1 discovery, critical gaps persist in harnessing its therapeutic potential. First, the clinical relevance of BECLIN-1 isoforms (e.g., BECLIN-1-Δexon11) remains undefined, with truncated variants demonstrating dominant-negative effects on autophagosome maturation in breast cancer models [12]. Second, post-translational modifications, like phosphorylation at Tyr133 by JAK2 and acetylation at Lys430/437, create dynamic “autophagy rheostats” that evade current biomarker assays. Third, the non-autophagic role of BECLIN-1 in STAT3-driven metastasis challenges the paradigm of autophagy-centric targeting.

This review synthesizes emergent insights from structural biology (particularly the domains for post-translational modifications and partner interactions), omics-based stratification, and preclinical models of BECLIN-1-targeted therapy to address these translational roadblocks. By reconciling molecular complexity with clinical realities, we chart a path toward context-aware therapeutic strategies that leverage BECLIN-1’s multifaceted biology beyond binary autophagy modulation.

## 2. History of *BECN1* Discovery at a Glance

The human *BECN1* gene consists of 11 introns and 12 exons encoding for 450 amino acids (aa) of the BECLIN-1 protein, with a molecular weight of 60 kDa [13]. The seminal work of Beth Levine’s group in 1999 precisely mapped the *BECN1* gene on the long arm of chromosome 17 (17q21) localized within 100 kb of the *BRCA1* locus, a well-known breast cancer susceptibility region frequently deleted in breast and ovarian cancers [14,15]. Sequence homology confirmed that *BECN1* is the mammalian ortholog of *Atg6*/*Vps30* in yeast. Importantly, restoring BECLIN-1 expression in *Atg6*/*Vps30* defective yeast or in *Becn1* mono-allelic deleted MCF7 breast cancer cells rescued autophagy and suppressed proliferation [13]. Subsequent in vivo studies demonstrated that *Becn1*^+/−^ mutant mice spontaneously develop tumors, including lymphomas, hepatocellular carcinoma, lung adenocarcinoma, and breast cancer, supporting the role of *BECN1* as a haploinsufficient tumor suppressor [16,17].

Additionally, in vivo and in vitro studies showed that mammary epithelial cells from Becn1^+/−^ mice were defective in clearance of apoptotic bodies (efferocytosis) during mammary involution, thus causing inflammation and deregulated mammary gland remodeling [18]. Later research revealed that BECLIN-1 localizes to the trans-Golgi network in complex with class III PI3K lipid kinase, or VPS34 [19]. Structural and biochemical studies further characterized two PI3K complexes (PI3KC): complex 1 (C1) with BECLIN-1, VPS34, ATG14, and VPS15, essential for autophagosome initiation, and complex 2 (C2) with BECLIN-1, VPS34, UVRAG, and VPS15, involved in autophagosome maturation [19,20,21,22].

Beyond its role as an autophagy scaffold, BECLIN-1 has emerged as a signaling hub that coordinates endo-lysosomal trafficking, receptor degradation, cell proliferation, and cell death through dynamic partner interactions [23,24,25,26,27].

## 3. Molecular Architecture and Interaction Landscape of BECLIN-1

**Figure 2:**
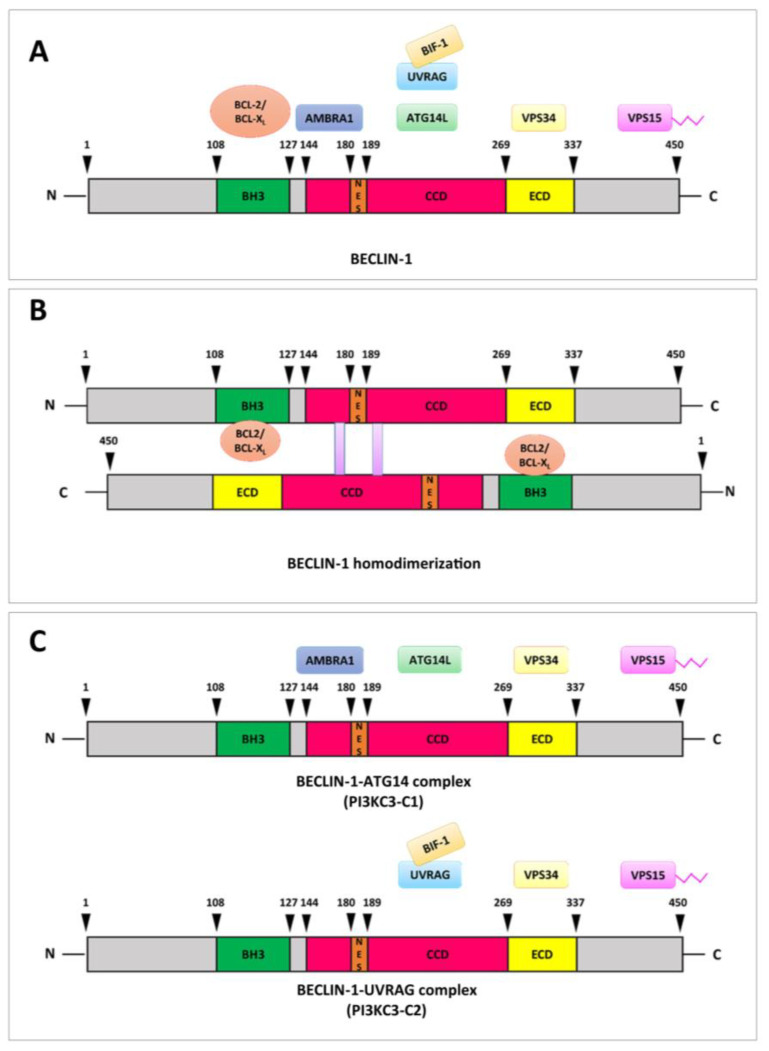
Domain architecture of BECLIN-1 coding sequence and its interaction landscape. (**A**) Structurally color-coded domains and their respective interactions: BH3 domain (green), CCD (magenta), ECD (yellow), and NES (orange). (**B**) The CCD can form metastable homodimers of BECLIN-1 that favors its binding to BCL-2/BCL-X_L_, contributing to the inactivation of BECLIN-1 pro-autophagy functions. This inactivation is reversible, as the metastable homodimers can readily transition to more stable heterodimeric complexes with partners like ATG14L or UVRAG, and (**C**) the two distinct PI3KC3 complexes: Complex I (PI3KC3-C1)—VPS34, VPS15, BECLIN-1, ATG14 and Complex II (PI3KC3-C2)—VPS34, VPS15, BECLIN-1, UVRAG, highlighting the dynamic nature of these complexes and the role of BECLIN-1 as a central scaffold in the initiation and the maturation of the autophagosome, respectively. Abbreviations: Activating molecule in BECLIN-1-regulated autophagy protein 1, AMBRA1; Autophagy-related gene, ATG; B-cell lymphoma 2, BCL-2; B-cell lymphoma-extra large, BCL-X_L_; BCL-2 homology 3, BH3; BAX-interacting factor-1, BIF-1; Coiled-coil domain, CCD; Evolutionary conserved domain, ECD; Nuclear export signal, NES; Phosphatidylinositol 3-kinase class III complex, PI3KC3-C; UV radiation resistance-associated gene, UVRAG; Vacuolar protein sorting, VPS.

The crystal structure of BECLIN-1 has revealed distinct domains involved in protein–protein interactions, asserting its role as a versatile scaffold for protein complexes with diverse cellular functions [28]. Figure 2 depicts the schematic overview of these domains and their interacting partners.

### 3.1. N-Terminal BH3 Domain (aa 108–127) and Beyond

This domain enables BECLIN-1 to interact with anti-apoptotic BCL-2 family proteins, such as BCL-2 and BCL-X_L_, thereby inhibiting autophagy [27,29]. Among various BCL-2 homologs, the Epstein–Barr virus protein BHRF1 requires not only the BH3 motif but also adjacent residues 90–170 for stable binding [30]. The N-terminal region of BECLIN-1 (aa 1–144) is the site of interaction with DIRAS3 (ARH-I), a Ras homolog with low intrinsic GTPase activity that acts as an imprinted tumor suppressor in ovarian cancers. GTP-bound DIRAS3 displaces BCL-2 and inhibits BECLIN-1 homodimerization, while simultaneously enhancing the interaction between BECLIN-1 and ATG14L, thereby promoting autophagy [31].

The BECLIN-1 BH3 domain is also involved in the interaction with TMEM9, an interactor of vacuolar ATPase necessary for lysosome acidification, to promote LC3-independent and Rab9-dependent alternative autophagy [32]. Finally, it is worth mentioning that the BH3 domain is involved in cytoplasmic p53-BECLIN-1 interaction that leads to BECLIN-1 ubiquitination and proteasome degradation, resulting in down-regulation of autophagy [33].

### 3.2. Coiled-Coil Domain (CCD; aa 174–266)

This domain is an interacting platform important for the autophagic and endocytic cofactors ATG14, AMBRA1, BIF1, UVRAG, and RUBICON [21,34,35]. ATG14 interacts with BECLIN-1 and VPS34 on the primary site of phagophore initiation and in the late stages of autophagosome biogenesis, when autophagosomes are mature and ready for fusion with endosomes and lysosomes [35,36,37]. A highly conserved flexible helical domain (FHD; aa 141–171) serves for binding AMBRA1, which is essential for initiating the starvation-induced autophagy [37]. The mutually exclusive competitive interactions between AMBRA1, BECLIN-1, and BCL-2 at the endoplasmic reticulum and at the mitochondrial membrane finely tune the crosstalk between pro-survival autophagy and apoptosis depending on the type and duration of the stress [38]. Under physiological conditions and in the absence of injuries, autophagy runs at a basal level for maintaining cellular homeostasis, whereas in response to stress, autophagy is induced as a tentative to face harmful damage and preserve cell viability [39]. In the first scenario, BCL-2 and NAF-1 (nutrient-deprivation autophagy factor-1) at the endoplasmic reticulum bind to BECLIN-1 to down-regulate autophagy while AMBRA1 is largely bound to BCL-2 at mitochondria, whereas in the second scenario, AMBRA1 relocates to the endoplasmic reticulum to displace BCL-2, promoting the formation of the BECLIN-1-PI3KC3 complex to initiate phagophore biogenesis [38] and at the same time freeing BCL-2 to prevent cell death [40]. UVRAG is critical for endosomal trafficking and interacts with BECLIN-1 and VPS34 (PI3KC3) in early and late endosomes [22]. At the same time, RUBICON acts as a negative regulator of autophagy and instead drives alternative endocytic pathways, such as LC3-associated phagocytosis (LAP) and LC3-associated endocytosis (LANDO), utilizing the autophagy machinery in a non-canonical manner [41]. Nutrient deprivation induces BIF1 to act as a sensor, interacting with ATG5 and LC3 on the emerging phagophore to promote autophagosome biogenesis. While the specific interaction site of BIF1 on BECLIN-1 is not explicitly detailed in the literature, it is known that UVRAG mediates their interaction. This BIF1-UVRAG-BECLIN-1 complex is functionally involved in autophagosome formation and, notably, plays a crucial role in receptor degradation and cytokinesis [42,43].

### 3.3. Nuclear Export Signal (NES; aa 180–189)

Initially described as a leucine-rich motif binding to chromosomal maintenance protein 1 (CRM1) for nuclear export, this domain was thought to be crucial for BECLIN-1-dependent autophagy and tumor suppressor functions [44]. However, recent studies have revealed nuclear functions of BECLIN-1 independent of autophagy. Intranuclear BECLIN-1 interacts with topoisomerase-II to facilitate DNA repair [45] and can regulate retinoblastoma (Rb) protein expression, thereby regulating cell cycle and cancer cell growth [46]. While earlier research emphasized the NES-mediated nuclear–cytoplasmic shuttling, current investigations suggest a more nuanced understanding of BECLIN-1 subcellular localization and functional diversity. The domain’s significance in promoting specific cellular processes remains an active area of research, challenging previous assumptions about its singular function in nuclear export.

### 3.4. C-Terminal Evolutionary Conserved Domain (ECD/BARA; aa 244–337)

This domain mediates the binding to membranes and the interaction with VPS34, triggering the production of the lipidic messenger PI3P necessary for autophagosome assembly [20]. This region also comprises three β-sheet–α-helix repeats and is therefore known as the β-α repeated autophagy-specific domain, or BARA domain [47]. VPS15, a large scaffolding protein (150 kDa), bridges and stabilizes the binding of VPS34 and ATG14 to BECLIN-1, anchoring the PI3KC3 complexes to membranes and favoring the recruitment of regulatory proteins [48,49,50]. The interaction between VPS15 and BECLIN-1 occurs via three key binding sites: the ECD, which overlaps with the BARA domain; the C-terminal residues 425–450, essential for membrane localization; and an intrinsically disordered N-terminal region that receives the majority of stimulating phosphorylation events. These interactions regulate the PI3KC3-C1 and C2 complexes, with the BECLIN-1-BARA domain interacting with the WD40 domain of VPS15 [50,51]. Notably, the 26 amino acids (425–450) at the C-terminal of BECLIN-1 play a fundamental role in its membrane localization and control of autophagosome biogenesis [52]. The tyrosine kinase adaptor protein GRB2 also binds BECLIN-1 ECD, linking HER2 signaling to autophagy regulation and potentially explaining how HER2 overexpression can influence autophagy and tumor progression [53].

## 4. Multilayered Regulation of BECLIN-1 in Cancer

**Figure 3:**
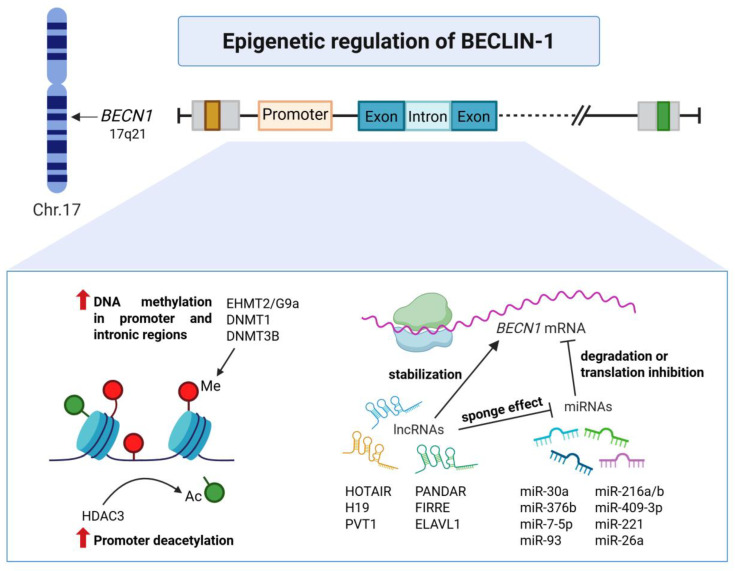
Epigenetic mechanisms regulating BECLIN-1 expression. Schematic representation of the epigenetic modifiers that control *BECN1* mRNA at transcriptional and post-transcriptional levels. The human *BECN1* gene (located on chromosome 17q21) undergoes several modifications, including DNA methylation and deacetylation, that result in reduced expression of BECLIN-1. Furthermore, *BECN1* mRNA translation is regulated by a subset of lncRNAs and miRNAs. The first ones can act as positive regulators either by stabilizing *BECN1* mRNA or by sponging miRNAs targeting BECLIN-1, while the latter ones are responsible for *BECN1* downregulation by eliciting mRNA degradation or translational inhibition. Abbreviations: Acetylation, Ac; DNA methyltransferase, DNMT; Histone deacetylase, HDAC; Euchromatic histone lysine N-methyltransferase 2, EHMT2; ELAV like RNA binding protein 1, ELAVL1; Functional intergenic repeating RNA element, FIRRE; Long non-coding RNAs, lncRNAs; H19 imprinted maternally expressed transcript, H19; HOX transcript antisense intergenic RNA, HOTAIR; Methylation, Me; MicroRNAs, miRNAs; promoter of CDKN1A antisense DNA damage activated RNA, PANDAR; Plasmacytoma variant translocation 1, PVT1.

Mutations in the *BECN1* gene are rare in human tumors [54]; however, monoallelic deletion of this gene occurs in ~75% of ovarian cancer and ~50% of breast cancer [14]. BECLIN-1 is tightly controlled by transcriptional, epigenetic, and post-transcriptional mechanisms, ensuring a fine balance between its tumor-suppressive and tumor-promoting roles depending on the microenvironmental context. Figure 3 schematically summarizes these regulatory layers.

### 4.1. Transcriptional Regulation

Several transcription factors directly bind the *BECN1* promoter to modulate its expression. The E2F family of transcription factors (E2F1, E2F2, and E2F3) activates *BECN1* transcription [55,56,57,58]. Depletion of E2F1-3 in osteosarcoma cells significantly reduces *BECN1* mRNA and protein levels, linking E2F activity to autophagy modulation [57].

NF-κB is a major regulator of inflammatory and immune responses and under stress (e.g., reactive oxygen species (ROS) or starvation) promotes *BECN1* transcription and pro-survival autophagy through its subunit RelA/p65 [59,60]. The inhibitors of apoptosis proteins (IAPs), X-linked IAP (XIAP), and cellular IAP1 (cIAP1) can activate NF-κB to induce *BECN1* transcription, a mechanism potentially linked to autophagy-mediated chemotherapy resistance in certain cancers [61]. Conversely, the protein TRIM59 acts as a negative regulator of the NF-κB pathway, attenuating *BECN1* transcription and down-regulating autophagy in non-small cell lung cancer (NSCLC) [62].

GA-binding protein, or GABP, has been shown to enhance BECLIN-1-dependent autophagy in triple-negative breast cancer cells by promoting *BECN1* transcription upon starvation, where forkhead box M1 (FOXM1) directly binds to the *BECN1* promoter [63,64]. In contrast, the transcription factor KLF5 (Krüppel-like factor 5), in cooperation with HDAC3 (histone deacetylase 3), can bind to and inhibit the *BECN1* promoter, leading to the suppression of *BECN1* transcription and autophagy in prostate cancer cells, potentially contributing to docetaxel resistance [65]. Other important transcription factors that can regulate *BECN1* are FOXO3a, FOS, XBP1, and STAT1, each responding to distinct cellular stressors through specific mechanistic pathways. FOXO3a was shown to bind the *BECN1* promoter and induce its transcription in hypoxic hepatocarcinoma cells [66]. FOS, as part of the AP-1 transcription factor complex, directly binds to a novel sequence (5’-TGCCTCA-3’) in the *BECN1* promoter in pheochromocytoma PC12 cells challenged with agonists [67]. In response to endoplasmic reticulum stress, the spliced isoform of XBP1 (X-box-binding protein 1) directly binds to the *BECN1* promoter region (nucleotides ranging from -537 to -755) in endothelial cells to induce its transcriptional activation and autophagy [68]. Conversely, during viral infection, STAT1 functions as a transcriptional suppressor of *BECN1* and other autophagy genes to avoid immune evasion, as demonstrated by CRISPR/Cas9 knockout studies showing significant upregulation of BECLIN-1, ULK1, and LC3 expression upon STAT1 depletion [69]. This integrated transcriptional network allows cells to fine-tune BECLIN-1 expression and autophagy activation in response to oxidative stress, hypoxia, neurotransmitter signaling, ER stress, and immune challenges, ensuring context-appropriate cellular homeostasis.

### 4.2. Epigenetic Regulation

The human *BECN1* gene contains a 1.5 kb CpG island from its 5’ end to intron 2 (nt -528 to 977), which can become aberrantly methylated in cancer. This highlights DNA methylation in the *BECN1* promoter and intronic regions as a silencing mechanism that correlates with its reduced expression in sporadic breast cancers [70]. Methylation in the promoter region by methyltransferases such as EHMT2/G9a and DNMT1 can repress the transcription of *BECN1* [70,71]. Other epigenetic regulators, such as the histone demethylase LSD1/KDM1A [72] and the acetyltransferases KLF5 and HDAC3 [65,73], also contribute to controlling transcript levels of the *BECN1* gene. BIX-01294, an EHMT2 inhibitor, can reverse the suppression of *BECN1* by allowing NF-κB and RNA polymerase II recruitment over the promoter region [71]. Krüppel-like factor 5 (KLF5), which is often deleted or hypoexpressed in prostate cancer, was shown to repress *BECN1* transcription [65]. Similarly, STAT3 represses *BECN1* repression in NSCLC by recruiting HDAC3 to its promoter [73].

### 4.3. Post-Transcriptional Regulation

#### 4.3.1. Micro-RNAs (miRNAs)

miRNAs act as post-transcriptional regulators by binding to complementary sequences in the 3’-untranslated regions (3’-UTR) of target mRNAs, promoting degradation or inhibiting translation. Several miRNAs have been found to modulate BECLIN-1 protein expression either by inhibiting the mRNA translation or degrading its mRNA and consequently modulating autophagy. Pioneering studies identified miR-30a and miR-376b as direct inhibitors of *BECN1* mRNA translation, preventing rapamycin- or starvation-induced autophagy in cancer cells [74,75]. Since then, other miRNAs, including miR-93, miR17-5p, and miR216a, have been shown to target *BECN1* mRNA and drive radiosensitivity in glioblastoma [76], pancreatic, and lung cancer cells [77,78]. The overexpression of miR-30a was shown to inhibit BECLIN-1-dependent autophagy and restore sensitivity to cisplatin and paclitaxel in gastric and lung cancer cells [79,80,81] as well as to the tyrosine kinase inhibitor imatinib in gastrointestinal stromal cells [82] and in chronic myeloid leukemia [83]. Suppression of BECLIN-1-dependent autophagy by miR-409-3p and miR-216b enhanced the efficacy of oxaliplatin [84] and of the *BRAF* (V600E) inhibitor vemurafenib [85], respectively. In breast cancer cells, down-regulation of BECLIN-1-mediated autophagy by miR-221 resulted in increased aggressiveness and resistance to cell death induced by IL-24 (as known as mda-7) [86]. Additional miRNAs, such as miR-26a and miR-124-3p, were found to target specifically the UTR of BECN1 mRNA in retinoblastoma and breast cancer cells, respectively [87,88].

It should be considered that some miRNAs can simultaneously target different ATG mRNAs, thus making it difficult to attribute the outcome solely to BECLIN-1 downregulation. For instance, the suppression of BECLIN-1 synthesis by miR-17-5p abrogated autophagy-dependent resistance to irradiation in glioma cells xenografted in nude mice [89]. However, this same microRNA was previously shown to target *ATG7* mRNA, leading to inhibition of autophagy and concomitant increased sensitivity to low-dose ionizing radiation in human glioblastoma cells [90].

Overall, these findings (summarized in Table 1) underscore the complexity of the miRNA-mediated regulatory network controlling BECLIN-1 and autophagy, with highly context-dependent implications for therapeutic response.

#### 4.3.2. Long Non-Coding RNAs (LncRNAs)

LncRNAs have emerged as crucial co-regulators of various biological processes for maintaining cellular homeostasis that, when altered, can become causative factors in the pathogenesis of several diseases, including cancer [91]. Although not translated into proteins, these RNA molecules play crucial roles in regulating gene activity, including the expression of the *BECN1* gene either directly or indirectly.

LncRNA H19 upregulates *BECN1* transcription by inhibiting S-adenosylhomocysteine hydrolase (SAHH), which decreases DNMT3B-dependent methylation of the *BECN1* promoter [92]. This was shown to increase BECLIN-1-dependent autophagy and resistance to tamoxifen in breast cancer cells [92].

LncRNAs also act as competing endogenous RNA (ceRNA), sponging miRNAs to modulate the expression of their targets. For example, lncRNA PVT1, frequently overexpressed in cisplatin-resistant lung cancer cells, sponges miR-216b, thereby upregulating BECLIN-1-dependent pro-survival autophagy [93].

Similarly, lncRNA HOTAIR functions as a ceRNA sequestering miR-17-5p to promote *BECN1* expression and enhancing autophagy-associated resistance to sunitinib in renal cancer cells [94]. Conversely, overexpression of lncRNA PANDAR in lung cancer promoted BECLIN-1 expression at both transcriptional and translational levels through still undefined mechanisms, thereby inhibiting cancer progression [95].

Furthermore, lncRNAs lead inter-regulatory pathways that involve numerous proteins such as the RNA-binding proteins (RBP) that can have a critical impact on the *BECN1* mRNA stability, transport, and overall expression dynamics [96]. The lncRNA FIRRE was found to interact and induce the translocation of polypyrimidine tract-binding protein (PTBP1) from the nucleus to the cytoplasm, stabilizing *BECN1* mRNA and thus promoting autophagy in colorectal cancer [97]. More recently, ELAVL1 (embryonic lethal abnormal vision-like RNA-binding protein 1) was found to stabilize lncRNA NEAT1, thereby increasing BECLIN-1-mediated autophagy and cancer progression in endometrial cancer cells [98].

In summary, emerging lncRNAs are key regulators of BECLIN-1, influencing autophagic activity and cancer progression through complex gene expression mechanisms. A deeper understanding of these pathways could provide important insights into the non-coding RNA-mediated control of autophagy with potential therapeutic implications.

#### 4.3.3. Alternative Splicing

**Figure 4:**
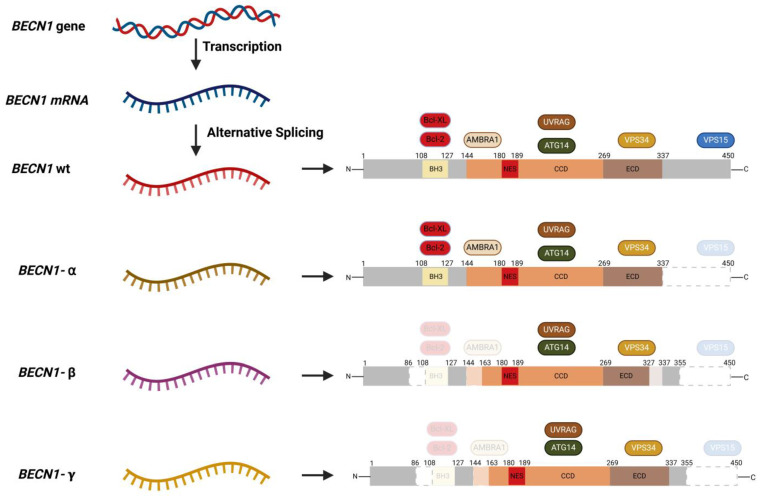
Alternative splicing of the *BECN1* mRNA. The *BECN1* mRNA undergoes alternative splicing to produce three BECLIN-1 splice variants (α, β, and *γ*). BECLIN-1-α, with a partial deletion in the C-terminal, hampers its interaction with VPS15, affecting its role in starvation-induced autophagy. BECLIN-1-β and -*γ* cannot interact with BCL-2 and AMBRA1 due to complete and partial deletions of the BH3 domain and CCD. The partial deletion of the ECD in BECLIN-1-β prevents it from interacting with VPS34, which negatively impacts autophagy. BECLIN-1-*γ*, however, retains its ability to bind VPS34 and only slightly affects autophagy. Abbreviations: Activating molecule in BECLIN-1-regulated autophagy protein 1, AMBRA1; Autophagy-related gene, ATG; B-cell lymphoma 2, BCL-2; B-cell lymphoma extra-large, BCL-X_L_; BCL-2 homology 3, BH3; Coiled-coil domain, CCD; Evolutionary conserved domain, ECD; Nuclear export signal, NES; UV radiation resistance-associated gene, UVRAG; Vacuolar protein sorting, VPS.

Alternative splicing is a process that results in various protein variants being produced from the same mRNA. This mechanism is particularly relevant in tumorigenesis, where it is often deregulated, and it is considered one of the hallmarks of cancer. Recent studies have shown that *BECN1* mRNA also undergoes alternative splicing to obtain isoforms with specific functions in autophagy. One first variant, named BECLIN-1s, has been identified in HeLa cells. In vitro experiments displayed that this variant, despite harboring a partial deletion of the ECD and the C-terminal domain, preserves the binding site for VPS34 and was shown to be able to drive the PARK2-dependent mitochondria-selective autophagy (mitophagy) process [99]. In our study [12], we corroborated the finding of splicing variant BECLIN-1-α lacking exon 11 in ovarian cancer cells, like what was observed by another group in human B-cell acute lymphoblastic leukemia cells [100]. The structural prediction of this variant pointed out a truncation (95 aa) in the C-terminal domain comprising the aromatic motif critical for VPS15 and VPS34 interaction that impaired starvation-mediated autophagy. In addition to BECLIN-1-α, two additional splicing variants, BECLIN-1-β and -ɣ, have also been identified in our study [12], which are graphically summarized in Figure 4. The first lacks the exons 5, 6, 10, and 11 that cause the deletion of the BH3 domain, losing the inhibitory site for BCL-2 interaction, and partially of the CCD and ECD, which makes the formation of the pro-autophagic complex weaker. Interestingly, the overexpression of this variant is enough to abrogate basal autophagy, suggesting a dominant negative effect on BECLIN-1 wild-type. Instead, the latter variant lacks the same exons of *BECN1* encoding BECLIN-1-β, except for exon 10. At the structural level, this variant loses the BH3 domain and 95 aa in the C-terminal domain, but it keeps an intact ECD. At the biological level, this variant is still prone to modulate basal autophagy, even with less efficiency compared to BECLIN-1 wild-type protein [12]. These findings support further studies to investigate the existence of *BECN1* mRNA splicing variants in other cancer model systems to understand their role in autophagy and endocytosis and explore their potential use as biomarkers in clinical settings.

## 5. Post-Translational Modifications on BECLIN-1

BECLIN-1 function is extensively fine-tuned by multiple post-translational modifications, including ubiquitination, acetylation, and phosphorylation. These modifications act as molecular switches that control its stability, subcellular localization, interactions with other proteins, and ultimately determine its activity [51,101].

### 5.1. Ubiquitination

Generally speaking, the stability of BECLIN-1 is under the control of the ubiquitination machinery. As mentioned above, the interaction with cytoplasmic p53 also drives BECLIN-1 to ubiquitination and proteasomal degradation [33].

The ubiquitin–proteasome system (UPS) is a complex and dynamic regulatory mechanism that modifies proteins through the attachment of ubiquitin molecules to certain lysine residues, forming various connections that determine unique functional outcomes.

Different ubiquitin linkages have opposite outcomes. This diversity of ubiquitin chain types provides multiple control mechanisms for BECLIN-1. K63-linked ubiquitination often facilitates autophagy by stabilizing BECLIN-1 and augmenting its contacts, whereas K48-linked ubiquitination generally directs BECLIN-1 towards proteasomal destruction, hence impeding autophagy [101]. Interestingly, the molecular chaperone HSP90 (heat shock protein 90) maintains BECLIN-1 stability, preventing K48-linked polyubiquitination [102]. Moreover, K11-linked ubiquitination has been identified as an alternative degradation signal affecting BECLIN-1 stability [103]. Particularly, NEDD4 (E3 ligase neural precursor cell expressed developmentally down-regulated protein 4) polyubiquitinates BECLIN-1 at K11, leading to its proteasomal degradation [104].

Researchers have identified multiple deubiquitinating enzymes (DUBs) that regulate the stability and function of BECLIN-1 and its consequent role in the autophagy process. Recently, USP24 was recognized as a pivotal regulator of autophagy-mediated ferroptosis, a mechanism of cell death that occurs in response to iron-dependent lipid peroxidation and inactivation of antioxidant responses [105], in hepatocellular carcinoma [106]. It was shown that USP24 delays BECLIN-1 degradation by specifically reducing its K48-linked ubiquitination, thereby promoting autophagy and ferroptosis to suppress tumor growth [106]. It is to be stressed, in this context, that ferroptosis is now recognized as a form of autophagic cell death where BECLIN-1 plays a major role [107].

Deubiquitinase USP5 stabilizes BECLIN-1 and enhances its nuclear accumulation, which inhibits p53-dependent senescence through MDM2-mediated p53 degradation. This USP5-BECLIN-1 axis is critical in overriding p53-dependent senescence in KRAS-driven tumorigenesis [108].

Other deubiquitinating enzymes, such as USP10, directly bind with BECLIN-1 to eliminate K48-linked ubiquitination, predominantly at Lys117 (K117) and Lys263 (K263), thus inhibiting its proteasomal destruction and facilitating autophagy. Conversely, USP13 selectively eliminates K63-linked ubiquitination, particularly at Lys402 (K402), hence influencing BECLIN-1 function in autophagic signaling and lysosomal transport [109]. Moreover, USP11 and USP14, which cleave K48, as well as USP19, which cleaves K437, have established contacts with BECLIN-1, thereby facilitating its stability and enhancing the regulation of autophagy [103,110,111,112]. Nonetheless, USP14, governed by AKT-mediated phosphorylation, inhibits autophagy by cleaving K63-linked ubiquitin chains from BECLIN-1, thus obstructing its association with the class III PI3K complex. USP14 functions as a dual regulator of autophagy and ubiquitin–proteasome system pathways [112]. Therefore, the differential yet complementary actions of these DUBs may highlight their crucial role in autophagic homeostasis, with their dysregulation implicated in cancer progression and other disorders [106,113].

The CUL3 (cullin 3) E3 ubiquitin ligase, in conjunction with the substrate adaptor KLHL38, facilitates K48-linked ubiquitination of BECLIN-1, resulting in its proteasomal destruction. Thus, elevated CUL3 expression inhibits autophagy and promotes tumor advancement and unfavorable prognosis in breast and ovarian malignancies [114]. Another E3 ligase, RNF216, facilitates K48-linked ubiquitination of BECLIN-1 in TLR-activated macrophages, hindering antimicrobial autophagy and advancing colon cancer progression [115].

The non-proteolytic ubiquitination of BECLIN-1 continues to be an area of active investigation. TRAF6 (E3-ligase tumor necrosis factor receptor (TNFR)-associated factor 6) promotes a non-proteolytic ubiquitination at lysine-117 (K117/Lys117) within the BH3 domain of BECLIN-1 and at lysine-63 (K63/Lys63) that is enhanced by Toll-like receptor-4 (TLR4) activation. On the contrary, the deubiquitinating enzyme A20 disrupts the interaction with BCL-2, promoting the inflammatory-induced autophagy by deubiquitinating the K63 [116].

AMBRA1 supports, with Rbx/Cul4-ligase, the polyubiquitination of K63, positively sustaining autophagy in response to starvation because of these ubiquitin chains acting as a scaffold for a stronger VPS34 interaction [109]. This mechanism is opposed by WASH protein that directly interacts with BECLIN-1, suppressing ubiquitination and autophagy [117].

### 5.2. Acetylation

The control of BECLIN-1 by acetylation depends mainly on the regulation of two residues, lysine-430 (Lys430) and lysine-437 (Lys437), that serve as critical regulatory nodes for autophagosome maturation, having implications spanning neurodegenerative disorders to cancer therapeutics. The acetyltransferase p300 catalyzes acetylation at both these residues, favoring RUBICON recruitment, thus inhibiting autophagosome assembly and endocytosis. On the contrary, NAD^+^-dependent deacetylase SIRT1 removes these acetyl groups, restoring autophagic flux and promoting autophagosome–lysosome fusion. This regulatory axis has been mechanistically linked to tumor progression, as acetylation-deficient BECLIN-1 mutants (K430R/K437R) enhance autophagosome maturation and suppress tumor growth in xenograft models [118].

The functional implications of BECLIN-1 acetylation are contingent upon the setting. In bladder cancer, SIRT1-mediated deacetylation of BECLIN-1 confers cisplatin resistance by preserving protective autophagy, which enables cancer cells to withstand the stress caused by chemotherapy. Preclinical data indicate that SIRT1 inhibition alleviates this resistance by enhancing BECLIN-1 acetylation, inhibiting autophagy, and reinstating cisplatin sensitivity in xenograft animals [119]. These findings underscore the therapeutic potential of targeting the SIRT1-BECLIN-1 axis to regulate autophagy in cancer therapy.

### 5.3. Phosphorylation

Most studies have been devoted to understanding phosphorylated sites mainly in the N-terminal and BH3 domain. Several of them have been demonstrated to be regulated in BECLIN-1 domains with a pro- or anti-autophagy role.

ULK1, the downstream target of mTOR, phosphorylates BECLIN-1 at serine-15 (Ser15), particularly when bound with ATG14 and VPS34, for initiating autophagy [120]. Moreover, the regulation at Ser15 is pivotal also for favoring the interaction of BECLIN-1 with PARK2 and its translocation on damaged mitochondria to mediate their specific degradation by mitophagy (see next Section 6.3) [121], while the phosphoglycerate kinase-1 (PGK1), critically involved in the production of ATP in the glycolytic pathway, regulates Ser30 in response to glutamine starvation or hypoxic environment [122].

The p38-downstream kinases MK2 and MK3 have as a target the serine-90 (Ser90), which is crucial to activate the pro-autophagic complex in response to starvation [123]. This same residue is also the target of two other kinases, DAPK3 and CaMKII (calcium/calmodulin-dependent protein kinase II), respectively demonstrated in cervical cancer and neuroblastoma cell lines [124]. In the latter model system, this phosphorylation enhanced the autophagic-mediated degradation of the protein Id1/2 (inhibitor of differentiation-1/2) to counteract cell differentiation [125].

BECLIN-1 has been found to be under control of the cell cycle checkpoint kinase 2 (CHK2), which is activated by ATM protein in response to DNA damage. Under oxidative stress and hypoxia, CHK2 phosphorylates BECLIN-1 at serine-90 (Ser90) and -93 (Ser93) to detach from BCL-2 and start autophagy (particularly mitophagy) to restore tissue homeostasis, a pathway that is impaired in CHK2^-/-^ knock-out mice subjected to cerebral ischemia [126]. AMPK is the main sensor of energy depletion, as it becomes active when ATP production is compromised. This kinase has two specific target sites on BECLIN-1, namely Ser93 and Ser96, that upon phosphorylation favor autophagy in response to glucose starvation [127]. Moreover, in response to glucose withdrawal, AMPK phosphorylates BECLIN-1 also in the C-terminal domain at threonine-388 (Thr388) to facilitate the assembly of the pro-autophagic complex [128]. Additionally, it has been shown that the interaction with ATG14 enhances AMPK-mediated phosphorylation of BECLIN-1 at Ser90 and Ser93 for maximal autophagy in response to starvation [54,127].

A great role in triggering autophagy is also played by DAPK-1/2- and ROCK-1 (Rho-associated coiled-coil containing protein kinase-1)-mediated phosphorylation of the BECLIN-1 BH3 domain, which promotes dissociation from BCL-2 [129,130,131,132]. On the other hand, several phosphorylations abrogate the pro-autophagic function of BECLIN-1. The pro-apoptotic kinase STK4/MST1 affects the activity of BECLIN-1 through phosphorylation at threonine-108 (Thr108) in the BH3 domain, allowing BAX-dependent apoptotic cell death [133].

Recently, the post-translational regulation of BECLIN-1 has been linked also to tumor microenvironment signals. Interleukin-6 (IL-6), one of the most common cytokines released by inflammatory and cancer cells, was shown to trigger JAK2-mediated phosphorylation of BECLIN-1 at tyrosine-333 (Tyr333), located in the region between BH3 and CCD, which promoted the interaction with VPS34, resulting in enhanced autophagy and chemoresistance in colon cancer [134].

Several tyrosine phosphorylation events in the ECD domain have inhibitory effects on autophagy. The human epidermal growth factor receptor (EGFR) has been well characterized to interfere with the BECLIN-1-dependent autophagy by phosphorylation at multiple sites in the ECD, namely tyrosine-229 (Y229), -233 (Y233), and -352 (Y352). In non-small cell lung carcinoma, such phosphorylation induced by EGFR stimulation inhibited autophagy while enhancing tumorigenesis and cancer progression [135]. Similarly, c-KIT and the non-receptor tyrosine kinase SRC have emerged as direct BECLIN-1 interactors that phosphorylate these critical tyrosine residues (Y229, Y233, and Y352). c-KIT physically associates with BECLIN-1 BH3 and ECD domains, forming an inhibitory complex that enhances BECLIN-1–BCL2 interactions while precluding BECLIN-1 from engaging the pro-autophagy PI3KC3 complex [136]. SRC employs a similar mechanism, binding to and phosphorylating BECLIN-1 at these sites to shift the protein into an inactive homodimeric state, thereby reducing VPS34 lipid kinase activity and blocking autophagic flux [137]. In breast cancer, EGFR complexes HER2 and BECLIN-1 at the plasma membrane to activate AKT and inhibit autophagy [138]. This inhibition provides an explanation of *HER2* amplification and suppressed autophagy and its mechanistic relevance in tumorigenesis sustained by oncogenic signals [35,139]. Nevertheless, it has been reported that breast cancer cells overexpressing ERBB2 (HER2) were tumorigenic and associated with autophagy suppression, similarly in wild-type and monoallelic deleted *BECN1* backgrounds [140].

Further, BECLIN-1 is regulated by the casein kinase 1 gamma 2 (CK1γ2) in the ECD at serine-409 (Ser409) that favors the binding with RUBICON to suppress the vesicular transport and autophagic response [118], and by the chimeric tyrosine kinase BCR-ABL in chronic myeloid leukemia, which both share the same target residue for phosphorylation that is tyrosine-233 (Tyr233), known to impair the interaction with ATG14 [141].

Even the oncoprotein AKT exerts a critical role in the control of BECLIN-1-dependent autophagy by phosphorylation of serine-234 (Ser234) and -295 (Ser295). This mechanism sequesters BECLIN-1 to vimentin and intermediate filaments by 14-3-3 protein, preventing the assembly of the pro-autophagic complex [142].

**Figure 5:**
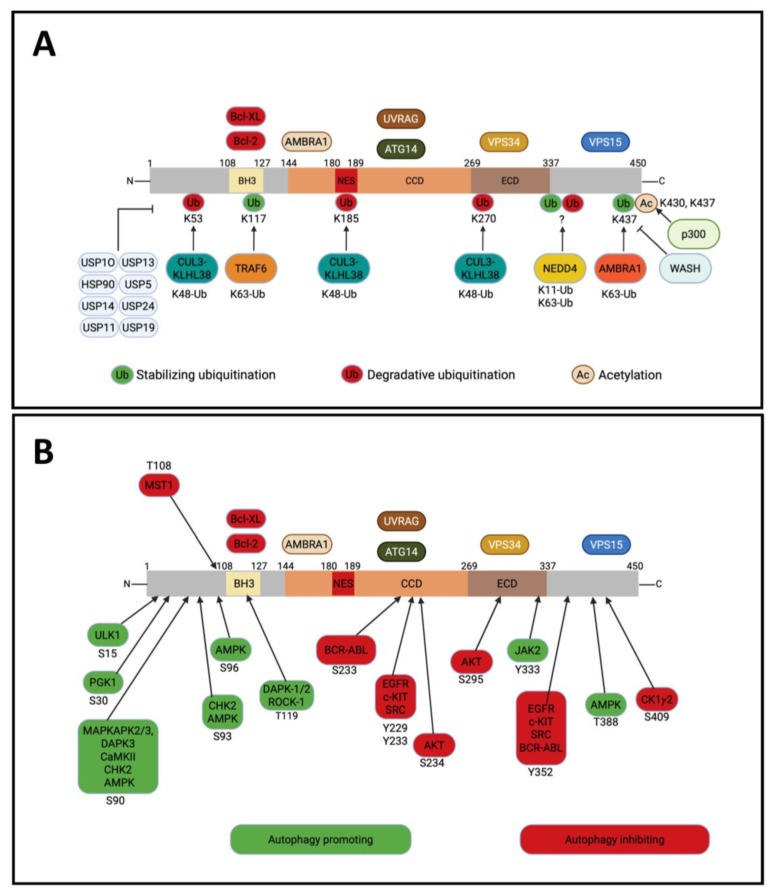
Site-specific post-translational modification sites on the BECLIN-1 amino acid sequence. The picture graphically represents the modifications related to ubiquitination, acetylation (**A**), and phosphorylation (**B**) of BECLIN-1 protein function. Activating or inactivating modifications are marked in green and red colors, respectively. Abbreviations: Acetylation, Ac; Activating molecule in BECLIN-1-regulated autophagy protein 1, AMBRA1; AMP-activated kinase, AMPK; Autophagy-related gene, ATG; B-cell lymphoma 2, BCL-2; B-cell lymphoma extra-large, BCL-XL; Breakpoint Cluster Region—Abelson Tyrosine Kinase, BCR-ABL; BCL-2 homology 3, BH3; Calcium/calmodulin-dependent protein kinase II, CaMKII; Casein kinase 1 gamma 2, CK1γ2; Checkpoint kinase 2, CHCK2; Coiled-coil domain, CCD; Cullin-3, CUL3; Death-associated protein kinase, DAPK; Evolutionary conserved domain, ECD; Epidermal growth factor receptor, EGFR; Histone acetyltransferase p300, p300; Janus kinase 2, JAK2; KIT proto-oncogene receptor tyrosine kinase, c-KIT; Kelch-like protein 38, KLHL38; Mitogen-activated protein kinase-activated protein kinase, MAPKAPK; Macrophage stimulating 1, MST1; Nuclear export signal, NES; Neural precursor cell expressed developmentally downregulated 4, NEDD4; Phosphoglycerate kinase 1, PGK1; Protein Kinase B, AKT; Proto-oncogene tyrosine-protein kinase, SCR; Rho-associated protein kinase 1, ROCK1; Tumor necrosis factor receptor-associated factor 6, TRAF6; Ubiquitination, Ub; Unc-51 like autophagy activating kinase 1, ULK1; Ubiquitin specific peptidase, USP; UV radiation resistance-associated gene, UVRAG; Vacuolar protein sorting, VPS; Wiskott-Aldrich syndrome protein and SCAR homologue, WASH.

Figure 5 illustrates the post-translational modifications that regulate BECLIN-1 stability and functions.

Taken together, these modifications illustrate the great molecular plasticity of BECLIN-1. Depending on the specific residues modified, BECLIN-1 may either promote survival by sustaining autophagy or trigger cell death by activating apoptosis, ferroptosis, or autophagic cell death.

## 6. Functional Role of BECLIN-1 in Cancer

### 6.1. Pivotal Link Between Autophagy and Apoptosis

BECLIN-1 occupies a pivotal position at the intersection of autophagy and apoptosis, exerting cellular responses to stress through its structural and functional plasticity. Notably, its BH3 domain functions as a rheostat that modulates the delicate balance between survival and death by enabling direct interactions with anti-apoptotic BCL-2 family members, like BCL-2 and BCL-XL [143]. Under nutrient-rich circumstances, BCL-2 binds BECLIN-1 to suppress autophagy, whereas under stress conditions this complex is disrupted by phosphorylation of BCL-2 (JNK1) or of BECLIN-1 itself (by DAPK), allowing BECLIN-1 heterodimerization with ATG14 and unleashing autophagy to mitigate metabolic stress [144].

Another layer of regulation comes from caspase-mediated cleavage of BECLIN-1 with generation of a C-terminal segment (BECLIN-1-C) that lacks autophagic activity and instead sensitizes cells to apoptotic signals. Translocation of BECLIN-1-C to the mitochondrial membrane enhances the BAX oligomerization, hence inducing the release of cytochrome c [145]. Cytoplasmic p53 is known to bind BECLIN-1 through the BH3 domain to direct its proteasomal degradation [33], yet it also binds to BCL-2 proteins through that very same domain to enhance mitochondrial permeabilization [146]. It seems that caspase-mediated cleavage of BECLIN-1 ensures that this C-fragment devoid of BH3 does not interfere with the pro-apoptotic function of p53 at the mitochondrial level.

Thus, BECLIN-1 functions as a molecular switch, facilitating pro-survival autophagy when intact and promoting apoptosis when cleaved by caspases [147,148,149].

In addition, the R-BiP/BECLIN-1/p62 complex was identified to play a crucial role in regulating the crosstalk between apoptosis and autophagy. Essentially, Ser 234/295 dephosphorylation of BECLIN-1 increased its cleavage and disruption of the R-BiP/Beclin-1/p62 complex, thus switching autophagy to apoptosis [150].

These results highlight the possibility of using the BECLIN-1-BCL-2 axis as a therapeutic tool to restore a balance between autophagy and apoptosis in cancer.

### 6.2. BECLIN-1-Dependent Selective Autophagy

Beyond bulk autophagy, BECLIN-1 also regulates selective forms of autophagy that influence cell fate. Selective autophagy exerts fine control of damaged organelle turnover in comparison to non-selective autophagy, which randomly degrades damaged materials inside the cells. Each organelle or molecule defines a specific subtype of autophagy, such as mitophagy (mitochondria), lysophagy (lysosomes), ER-phagy (endoplasmic reticulum), ribophagy (ribosomes), aggrephagy (protein aggregates), or xenophagy (pathogens) [151,152].

For target-specific autophagy, the cargo to be degraded must be specifically labeled. The most widely studied cargo labeling involves p62, or sequestosome-1, an autophagy receptor that plays a role in the ubiquitin system. As a result of its interaction with LC3, it sequesters ubiquitinated proteins within autophagosomes that are then delivered to lysosomes for degradation [153]. In particular, BECLIN-1 is well-characterized to regulate mitophagy and lysophagy, the two major pathways that have significant consequences for cancer progression and treatment resistance, by interacting dynamically with complexes associated with autophagy and by undergoing post-translational changes.

### 6.3. Mitophagy

Mitophagy is a selective form of autophagy that is essential for eliminating depolarized or damaged mitochondria, thereby maintaining the quality of mitochondria [154]. Metabolic waste products, including those generated by fatty acid oxidation, the Krebs cycle, and oxidative phosphorylation (OXPHOS) [155], can be detrimental to cellular fitness by promoting the oxidation of proteins, nucleic acids, and lipids, and by stimulating cell death pathways [156,157]. BECLIN-1 is indispensable for Parkin-mediated mitophagy. PINK1, a serine/threonine kinase, acts as a sensor for damaged mitochondria by working with the mitochondrial membrane complex TOM20 [158]. Upon activation, Parkin, a ubiquitin ligase, is then recruited to the mitochondria membrane, and mitophagy can start [159,160,161]. Here, both PINK1 and Parkin can interact directly with BECLIN-1 to trigger autophagosome formation at the mitochondria-ER contact site and shape the omegasome through ULK-1-mediated active phosphorylation of BECLIN-1 at Ser15 [162,163,164,165]. In addition to BECLIN-1, Parkin also interacts with AMBRA1, which facilitates the BECLIN-1-VPS34-dependent assembly of autophagosomes incorporating depolarized mitochondria through the LC3-p62 interaction in the perinuclear clusters [166]. Notably, BECLIN-1-deficient cells exhibit defective mitochondrial sequestration despite Parkin recruitment, underscoring its non-redundant role in autophagosome–mitochondria tethering [164].

### 6.4. Lysophagy

After partial or complete rupture of lysosomes, if repair mechanisms fail, these organelles become ubiquitinated and are degraded through selective autophagy, also known as lysophagy [167]. Lysophagy is typically carried out by a TRIMs-galectin-3-dependent pathway, in which galectin marks damaged lysosomes and ensures their degradation through autophagosome [168], in a BECLIN-1-dependent manner [169]. For successful TRIMs interaction, two BECLIN-1 domains are strictly required, one located between BH3 and CCD, and the other overlapping with the ECD [170]. Along with BECLIN-1, ATG16L is recruited to the marked lysosomes, linking the pro-autophagic complex PI3KC3 with the proteins WIPI2 and LC3 [169]. Recent work demonstrates that BECLIN-1 interaction with TMEM9, an endolysosomal regulator, activates Rab9-dependent alternative autophagy pathways during lysosomal stress—a process hijacked by cancer cells to evade lysosome-targeted therapies [32].

### 6.5. BECLIN-1 in Endocytotic Trafficking and Receptor Signaling in Cancer

Endocytosis is an essential cellular process governing the uptake, trafficking, and degradation of molecules, including receptors, that regulate cell signaling and homeostasis [171]. Being the core component of the class III phosphatidylinositol 3-kinase complex together with UVRAG (PI3KC3-C2), BECLIN-1 participates in endocytic trafficking. Fundamentally, PI3KC3-C2 leads the endocytic trafficking, lysosomal maturation, and cytokinesis by generating phosphatidylinositol-3-phosphate (PI3P) on intracellular membranes [43,172,173]. Although the relevance of PI3KC3-C2 in autophagy is contentious, its essential functions in membrane trafficking and endosomal sorting are firmly established.

BECLIN-1 depletion impairs retrograde transport of endosomes to the Golgi compartment, affecting organelle trafficking and neuronal development in *C. elegans* and mouse models [173,174]. Additionally, BECLIN-1 regulates early endosome maturation in coordination with UVRAG and VPS34, transitioning endosomes from APPL1+/PI3P− to PI3P+ states. These PI3P+ endosomes recruit EEA1 to facilitate fusion and maturation via Rab5 and CMTM7, enabling proper EGF trafficking and receptor degradation [175,176,177,178,179].

PI3P produced by PI3KC3-C2 is recruited by activated Rab5 through VPS15, facilitating the anchoring and fusing of early endosomes [180]. During endosomal maturation, Rab7 supplants Rab5, facilitating the interaction of PI3KC3-C2 with late endosomes through VPS34 and VPS15 [181]. This maturation supports the retromer complex and ESCRT machinery, which recycle cargo proteins like GLUT1 to the plasma membrane or trans-Golgi network and sort ubiquitinated receptors like EGFR into intraluminal vesicles for lysosomal degradation [182,183].

BECLIN-1 tightly regulates the tumor growth and progression, imparting its tumor suppressive effect via endo-lysosomal trafficking, a process that is crucial in controlling the cell surface receptor function. Recently, it was shown that BECLIN-1 promoted the endosomal recruitment of hepatocyte growth factor-regulated tyrosine kinase substrate (HRS), enabling sorting of EGFR and transferrin receptor (TFR1) into intraluminal vesicles for lysosomal degradation. Loss of BECLIN-1 in breast cancer cells reduces HRS recruitment, prolonging EGFR/ERK signaling and TFR1-driven iron uptake, ultimately supporting tumor growth. This pathway is independent of autophagy, as ATG5 or ATG7 deletion does not emulate similar consequences [184]. In congruence, BECLIN-1 loss increased AKT/ERK activation and promoted breast carcinoma invasion by prolonging growth factor receptor retention in signaling compartments enriched with PI3P/APPL1 [177].

Moreover, BECLIN-1 is essential for preserving epithelial structure through the regulation of E-cadherin trafficking. The PI3KC3-C2 complex facilitates the appropriate recycling of E-cadherin to the plasma membrane and inhibits its lysosomal breakdown. The disruption of BECLIN-1 destabilizes E-cadherin localization at adherent junctions, hence activating β-catenin/Wnt signaling and promoting epithelial–mesenchymal transition [24,185]. A recent work involving intestinal epithelial cells revealed that *BECN1* deletion results in the cytoplasmic mislocalization of E-cadherin and occludin, hence affecting apical F-actin organization and epithelial polarity [24]. In breast cancer models, BECLIN-1 overexpression reinstates plasma membrane E-cadherin levels, inhibiting mesenchymal markers like Snail and Zeb1 [185].

Under starvation or exposure to tyrosine kinase inhibitors (TKI), like erlotinib or gefitinib, EGFR signals are blocked by the detachment of RUBICON from BECLIN-1, mediated by the endosome-associated protein LAPTM4B (lysosomal-associated protein transmembrane 4 beta) [186,187]. These mechanisms illustrate BECLIN-1’s multifaceted role in receptor signaling regulation, endocytic maturation, and tumor suppression via trafficking pathways.

### 6.6. BECLIN-1 Crosstalk with Other Pathways in Cancer

The PI3K/AKT/mTOR pathway is amongst the major oncogenic cascades for cellular growth and metabolism and is paralleled by inhibition of BECLIN-1-dependent autophagy [188]. Studies have shown that the inflammatory cytokine interleukin-7 (IL-7) activates the PI3K/AKT/mTOR signaling pathway, thereby downregulating BECLIN-1 expression significantly in the lung cancer cells. This reduction of autophagy creates a tumor-favorable environment by promoting proliferation and survival while suppressing the apoptotic responses [189]. This bidirectional relationship has significant implications for cancer therapy. For instance, LTX-315, a polypeptide that increases BECLIN-1 levels and alters p-AKT and p-mTOR levels, has shown promise in enhancing drug responsiveness in ovarian cancer models [190]. Similarly, Xie–Bai–San (XBS) regulates both mTOR and BECLIN-1, inhibiting gefitinib-induced autophagy in non-small cell lung cancer cells and promoting cell death by enhancing p-mTOR and BCL-2 levels while decreasing BECLIN-1 levels [191]. Additionally, endoplasmic reticulum-mediated regulation of BECLIN-1 adds another layer of complexity to autophagy control.

Beyond the canonical mTOR-dependent regulation, BECLIN-1 has been found to interact with GRB2 through the BECLIN-1 ECD domain and results in negative regulation of autophagy by modulating the activity of VPS34. Notably, BECLIN-1 interaction with GRB2 represents a distinct regulatory pathway that bypasses traditional PI3K/AKT/mTOR signaling, suggesting that BECLIN-1 autophagy regulatory capacity extends beyond well-characterized protein partnerships [53].

Notable interactions of BECLIN-1 also include activation of the STAT3 signaling pathway independently of its known role in autophagy. Mechanistically, BECLIN-1 regulates the interaction between STAT3 and JAK2, which, when knocked down, promotes STAT3 phosphorylation through enhanced STAT3-JAK2 interaction. Low expressions of BECLIN-1 directly correlated with more aggressive phenotypes of colorectal cancer with high cell motility and invasion [192]. Importantly, this regulatory function of BECLIN-1 suggests additional non-canonical functions of this protein in cancer progression.

The interaction between BECLIN-1 and anti-apoptotic BCL-2 family proteins represents another critical regulatory node, which is modulated by several proteins. One of these proteins is LETM1, a protein playing a major role in mitochondrial homeostasis. Depletion of LETM1 triggers AMPK-mediated phosphorylation of BCL-2, causing disruption of the BECLIN-1/BCL-2 complex and promoting both apoptosis and autophagy in liver cancer cells [193].

Apparently, recent discoveries have expanded the role of BECLIN-1 beyond apoptosis and autophagy to include other cell death pathways. Key phosphorylation of Ser90, Ser93, and Ser96 on BECLIN-1 by active AMPK leads to the formation of a BECLIN-1-SLC7A11 complex, which directly inhibits system Xc− activity and ultimately promotes ferroptosis, an iron-dependent form of programmed cell death [194]. Paradoxically, BECLIN-1 serves as an inhibitor of necroptosis by associating with phosphorylated MLKL in the necrosome complex, preventing MLKL oligomerization and subsequent necroptotic cell death. Such findings put forth the BECLIN-1 complex’s role in regulating multiple cell death pathways, with significant therapeutic implications [195]. Moreover, BECLIN-1 also mediates autosis, a morphologically distinct form of autophagic cell death governed by Na^+^/K^+^-ATPase activity and characterized by focal ballooning of the perinuclear space due to accumulation of autophagosomes. Experimental evidence in pancreatic cancer cells demonstrates that peptide-based BECLIN-1-targeting perturbation can induce excessive autophagy that triggers autosis-like features, including mitochondrial stress and non-apoptotic cell death [196].

The interaction between BECLIN-1 and mitogen-activated protein kinase (MAPK) pathways, including ERK, JNK, and p38, represents another important regulatory network. Allyl isothiocyanate has been shown to trigger ERK, AMPK, and JNK signaling pathways and to increase BECLIN-1-dependent autophagy [197].

## 7. Clinical Implications and Biomarker Potential

### 7.1. Prognostic, Predictive, and Diagnostic Value of BECLIN-1 Across Cancer Types

The clinical impact of BECLIN-1 reflects its complex and context-dependent role in tumor biology. Its expression level has been investigated in patient samples through a standardized assessment with immunostaining methodologies, with immunohistochemistry (IHC) on formalin-fixed and paraffin-embedded tissues serving as the primary approach for protein-level detection [198], complemented by transcript-level measurements (qRT-PCR, microarray, or RNA-seq) and ELISA detection in fluid samples. The results, however, are not always uniform, underscoring the multifaceted functions of this protein in different tumor types and disease stages. Low expression of BECLIN-1 has been reported in breast cancer [199], ovarian cancer [200,201,202], lung cancer [203,204], cholangiocarcinoma [205,206], gastric cancer [207], and colorectal cancer [192,208], compared to the adjacent normal tissue, suggesting that defective autophagy may favor carcinogenesis, metastatic phenotype, and worse prognosis.

In detail, low levels of BECLIN-1 were found associated with the most aggressive pathological traits and linked to poor overall survival in a cohort of estrogen-receptor (ER)-negative subtypes of breast cancer, as observed in published datasets TCGA METABRIC [54]. Similarly, in non-Hodgkin lymphomas and ovarian cancer, elevated BECLIN-1 expression is linked to less aggressive tumors and improved chemotherapy response, contributing to better survival outcomes [209,210]. Further, findings from our group corroborated these observations in diffuse large B-cell lymphoma, where high expression of BECLIN-1 may enhance chemotherapy responsiveness through increased autophagy, making it a valuable prognostic factor correlated with longer survival [211].

However, despite BECLIN-1 establishing value as a pro-survival marker and therapeutic target, its role in cancer is more nuanced. Several studies sternly indicate contrasting patterns in breast, gastric, endometrial adenocarcinoma, and colorectal cancer, where the elevated BECLIN-1 levels have been anomalously associated with patient bad prognosis as well as the aggressive phenotypes, nodal metastasis, and therapy resistance [212,213,214]. These studies signify that the expression and function of BECLIN-1 are tightly interwoven with clinical outcomes across multiple tumor types and that its role can vary across different tumor types and contexts.

In addition to BECLIN-1, circulating molecular markers, like LC3 and p62, can be integrated into prognostic indexes to further refine patient stratification. For instance, our recent in silico TCGA analysis of the AML cohort revealed that high expression of BECLIN-1, in conjunction with high MAP1LC3B and low p62/SQSTM1 levels (indicative of high autophagy flux), correlated with better prognosis. AML patients with high autophagy–mitophagy signatures driven by BECLIN-1 exhibited the longest overall survival, reinforcing BECLIN-1’s pivotal role in regulating autophagic processes that positively influence clinical outcomes [215].

Moreover, in colorectal cancer, phosphorylation of BECLIN-1 at Y333 by SRC kinase suppresses autophagy, facilitating chemoresistance and establishing it as a predictive marker of therapeutic response [134]. Concomitantly, BECLIN-1 expression in cholangiocarcinoma not only associates with increased autophagy and better patient survival, but its upregulation by the nutraceutical resveratrol also promotes apoptosis, suppresses tumor cell proliferation, and inhibits the pro-tumorigenic IL6/IL6R signaling axis [216].

In hepatocellular carcinoma, serum BECLIN-1 levels distinguish cirrhotic patients with malignancy, while co-evaluation with ATG5 and cachexia scoring improves early detection and risk stratification, highlighting its utility not only as a molecular indicator but also as a potential therapeutic target [217].

Overall, these findings firmly establish BECLIN-1 as a biomarker for prognosis, diagnosis, and therapy response prediction, with clinical implications shaped by tumor type, subtype, and molecular context.

### 7.2. Therapeutic Targeting of BECLIN-1 Dependent Pathways

Recent advances in understanding the pleiotropic roles of BECLIN-1 have positioned its targeted activation as a promising strategy to overcome therapeutic resistance and reshape immunosuppressive tumor microenvironments. By prioritizing pharmacological and genetic approaches to enhance BECLIN-1-dependent autophagy, researchers are uncovering novel mechanisms to amplify anti-tumor immunity while suppressing pro-metastatic signaling pathways.

Innovative therapeutic strategies aimed at the BECLIN-1–BCL-2 axis and the pharmacological activation of autophagy through cell-permeable peptides are gaining momentum. Typically, these peptides contain the N-terminal of the Tat sequence and a hydrocarbon staple-modified fragment of the target protein. One such peptide, Tat-SP4 (stapled peptide 4), bearing a fragment targeting the BECLIN-1 C-terminal, was shown to inhibit pancreatic cancer cell proliferation by inducing excessive autophagy, lysosomal degradation of EGFR, and significant mitochondrial stress [196]. Peptides like Tat-SP4 and small molecules such as compound 35, which are further engineered to selectively disrupt BECLIN-1/BCL-2 interactions without affecting pro-apoptotic pathways, exemplify the potential to precisely modulate autophagy to induce cancer cell death while maintaining normal cellular function [218]. Similarly, the Tat-BECLIN-1 peptide restores autophagic death in HER2+ breast cancer [139], BAP1-deficient clear renal cell carcinoma [137], and Merkel cell carcinoma [136]. In BAP1-mutant cancers, SRC inhibitors (namely, dasatinib, bosutinib, and saracatinib) synergize with autophagy inducers Tat-BECLIN-1, SW076956, and SW063058 to restore BECLIN-1-mediated autophagy and suppress tumor growth both in vitro and in ovo, and in patient-derived organoids of uveal melanoma and clear cell renal carcinoma [137].

BECLIN-1’s role in immune regulation and tumor suppression underscores its therapeutic potential when strategically induced. Studies have shown that myeloid-specific BECLIN-1 deficiency leads to neutrophilia, aberrant p38 activation, and immunosuppressive interactions between neutrophils and pre-B cells via the CXCL9/CXCR3 chemotaxis pathway. These interactions elevate the expression of PD-L1 and IL-10, suppressing CD8+ T cell function and promoting lymphoma. Pharmacological or genetic activation of BECLIN-1 could disrupt these pro-tumorigenic networks by preventing p38 hyperactivation and restoring immune surveillance. Preclinical models indicate that BECLIN-1-inducing agents can reduce PD-L1 expression by 60%, enhancing CD8+ T cell cytotoxicity against tumor cells [219].

Targeting BECLIN-1-mediated autophagy has been shown to disrupt immune suppression in solid tumors. Genetic inhibition of *BECN1* in melanoma models enhances NK cell infiltration via CCL5 upregulation, impairing tumor growth [220]. In gastric adenocarcinoma, *BECN1* activation correlates with improved outcomes, with high BECLIN-1 expression linked to increased FOXP3+ Treg infiltration, smaller tumors, reduced metastasis, and enhanced CD8+ T cell activity. Nanoparticle systems co-delivering *BECN1* mRNA with IL-2 are being explored to regulate Treg recruitment while sustaining anti-tumor immunity [221].

Beyond direct tumor suppression, innovations highlight BECLIN-1’s broader therapeutic relevance. Metabolic reprogramming of CAR-T cells using autophagy-inducing agents such as semaglutide and Urolithin A enhances CAR-T cell persistence, memory, and anti-tumor efficacy, indirectly leveraging BECLIN-1-mediated autophagy [222]. The evidence that GLP-1R agonists and Urolithin A can collaboratively stimulate autophagy and mitophagy in CAR-T cells provides a foundation for combining metabolic modulation with immunotherapy [222]. These techniques may augment cytotoxic immune responses, especially in refractory or metastatic cancers [223].

The therapeutic activation of BECLIN-1 also opens avenues for combination strategies with existing modalities. For example, BH3 mimetics such as ABT-737 can liberate BECLIN-1 from inhibitory interactions with anti-apoptotic BCL-2 proteins, promoting autophagy. This induction can sensitize cancer cells to apoptosis when modulated appropriately [224,225,226,227]. Additionally, agents like metformin bidirectionally regulate autophagy, promoting autophagic cell death in some cancers while suppressing it in others, presenting a dual regulatory role [228,229,230,231]. Alternatively, preclinical studies on Spautin-1, a USP10/13 inhibitor, show promise in stabilizing BECLIN-1 and reducing K48-linked ubiquitination in glioblastoma and NSCLC patients with BECLIN-1 promoter methylation [232,233].

**Figure 6:**
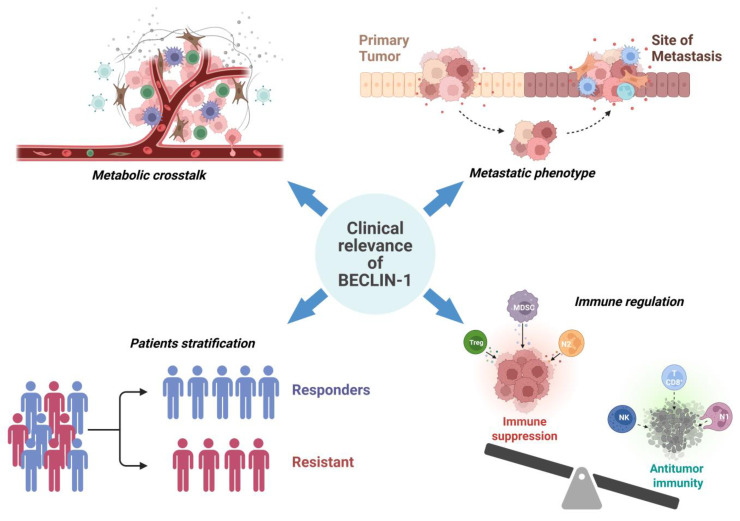
Clinical relevance of BECLIN-1 expression in cancer. Schematic representation of the main implications of BECLIN-1 in terms of prognostic value and regulation of cancer hallmarks (e.g., metabolic crosstalk in the microenvironment, metastatic behavior, and regulation of anti-cancer immunity). The illustration highlights that BECLIN-1 expression can help in stratifying cancer patients into responder vs. non-responder groups to predict the therapeutic outcomes. This prognostic relevance can be attributed to the functional role of BECLIN-1 in the modulation of pro-metastatic, pro-tumorigenic signaling pathways and the immunosuppressive landscape. Abbreviations: N1, type 1 neutrophils (anti-tumor); N2, type 2 neutrophils (pro-tumor); NK, natural killer cells; T CD8+, T-cells; MDSCs, myeloid-derived suppressive cells; T regs, regulatory T-cells.

Taken together, these findings position BECLIN-1 as not only a tumor suppressor and diagnostic–prognostic marker but also a valuable target for designing context-specific autophagy-modulating anti-cancer therapies. Obviously, before entering the clinic, it will be mandatory to carefully evaluate the possible side effects resulting from the chronic use of these therapies. Figure 6 summarizes the main translational implications of BECLIN-1 described above.

## 8. Conclusions and Future Prospectives

BECLIN-1 has become a pivotal regulator of autophagy, closely connecting cellular survival, death, and metabolism. Its complex significance is highlighted by several regulatory mechanisms, including genetic and epigenetic control of its transcription, alternative splicing, extensive post-translational modifications, and interactions with essential signaling molecules, which together influence tumor growth and therapeutic response. Epigenetic regulators represent a cutting-edge therapeutic tool. In this context, it is interesting to note that many natural products (particularly nutraceuticals such as resveratrol and curcumin) exert antitumor effects also through epigenetic regulation of BECLIN-1-dependent autophagy [234]. Future research should focus on clarifying the functions of BECLIN-1 splice variants in autophagy and endocytosis, enhancing our comprehension of dynamic post-translational modifications to discover new targets for selective autophagy activation, and formulating combination therapies that merge BECLIN-1-targeted agents with current chemotherapeutic, immunotherapeutic, or metabolic modulators. In this regard, it is fundamental to determine whether splicing of *BECN1* mRNA also occurs in normal cells and in which situations and what could be the physiological function of the spliced isoforms.

Overall, a thorough understanding of the regulatory networks governing BECLIN-1 will enhance the strategies for autophagy-based cancer therapies and perhaps facilitate innovative treatments for other pathologies. By coupling structural insights with translational research, BECLIN-1 emerges as a prospective focal point for the advancement of precision therapeutics that harness the full potential of autophagy modulation.

## Figures and Tables

**Table 1:** miRNAs targeting BECLIN-1 along with the experimental model used for the research findings.

miRNA	Experimental Model	Reference
miR-30a	Breast cancer (in vitro) Glioblastoma (in vitro) Lung cancer (in vitro) Gastric cancer (in vivo and in vitro) Hepatocellular carcinoma (in vitro and in vivo) Ovarian cancer (in vitro and in vivo) Gastrointestinal stromal tumors (in vitro and in vivo) Chronic myeloid leukemia (in vitro)	[74] [79] [81] [82] [83]
miR-376b	Breast cancer (in vitro) Hepatocellular carcinoma (in vitro)	[75]
miR-17-5p	Glioma (in vitro and in vivo)	[89]
miR-216a	Pancreatic Cancer (in vitro and in vivo)	[77]
miR-93	Glioblastoma (in vitro and in vivo) Lung cancer (in vitro) Melanoma (in vitro)	[76]
miR-409-3p	Colorectal cancer (in vitro and in vivo)	[84]
miR-216b	Melanoma (in vitro and in vivo)	[85]
miR-221	Breast cancer (in vivo and in vitro) Lung cancer (in vitro) Prostate cancer (in vitro)	[86]
miR-26a	Retinoblastoma (in vitro)	[87]
miR-124-3p	Breast cancer (in vivo and in vitro)	[88]

## Data Availability

No new data were created in this manuscript. Illustrations are original artwork prepared by the authors.

## Appendix A. Beclin-2

BECLIN-2 was discovered by Beth Levine’s lab in 2003 as a novel autophagy-related protein homologous to BECLIN-1 with a propensity to degrade several G protein-coupled receptors (GPCRs) with GASP1. The *BECN2* gene is located on chromosome 1q43 and shares only 57% of sequence homology with *BECN1*. In humans, it transcribes a protein with 431 aa and a predicted molecular weight of 49 kDa, with the same functional domains as BECLIN-1 [235]. Unlike *BECN1*, the deletion of *BECN2* (*BECN2*^-/-^) does not affect embryonic development and birth in mice but enhances tissue alterations such as splenomegaly, lymphadenopathy, and diffuse inflammatory state [235,236]. BECLIN-2 has been associated with the inhibition of the inflammatory pathway NF-ΚB through autophagic degradation, independent of BECLIN-1, of MEKK3 and TAK1, which are included in ATG9^+^-vesicles that fuse with autophagosomes thanks to interactions with STX-5 and -6 [236]. BECLIN-2 also negatively controls inflammasome activation and directly interacts with component proteins of this complex, such as NLRP3 and NLRP1, AIM2, NLRC4, and CASP1, through its CCD-ECD domain. These proteins are degraded by lysosomes with the previously described mechanism involving ATG9^+^ vesicles [236]. Recent observations confirm the common role for BECLIN-1 and BECLIN-2 in promoting the formation of LC3^+^-autophagosomes but a divergent role in mitophagy, where only BECLIN-1 appears necessary, and unexpectedly, the degradation of mitochondria is enhanced in cells lacking BECLIN-2 [165]. Furthermore, BECLIN-2 has been described as unable to interact with RUBICON, and it does not require the detachment of BCL-2 to mediate the starvation-induced autophagy because it lacks the functional residue (Thr119) controlled by DAPK1. However, it displays interactive ability with ATG14, VPS34, and AMBRA1, like BECLIN-1 [235,237].
